# Nonaqueous Polyethylene Glycol as a Safer Alternative to Ethanolic Propolis Extracts with Comparable Antioxidant and Antimicrobial Activity

**DOI:** 10.3390/antiox10060978

**Published:** 2021-06-18

**Authors:** Jelena Šuran, Ivica Cepanec, Tomislav Mašek, Kristina Starčević, Ivana Tlak Gajger, Mihaela Vranješ, Božo Radić, Saša Radić, Ivan Kosalec, Josipa Vlainić

**Affiliations:** 1 Department of Pharmacology and Toxicology, Faculty of Veterinary Medicine, University of Zagreb, Heinzelova 55, 10000 Zagreb, Croatia; 2 Amelia Ltd., Zagorska 28, Bunjani, 10314 Kriz, Croatia; ivica.cepanec@amelia.hr; 3 Department of Animal Nutrition and Dietetics, Faculty of Veterinary Medicine, University of Zagreb, Heinzelova 55, 10000 Zagreb, Croatia; tomislav.masek@vef.hr; 4 Department of Chemistry and Biochemistry, Faculty of Veterinary Medicine, University of Zagreb, Heinzelova 55, 10000 Zagreb, Croatia; kristina.starcevic@vef.hr; 5 Department for Biology and Pathology of Fish and Bees, Faculty of Veterinary Medicine, University of Zagreb, Heinzelova 55, 10000 Zagreb, Croatia; ivana.tlak@vef.hr; 6 Faculty of Veterinary Medicine, University of Zagreb, Heinzelova 55, 10000 Zagreb, Croatia; mihaela.vranjes@hotmail.com; 7 Hedera Ltd., 4. Gardijske Brigade 35, 21311 Split, Croatia; bozo@hedera.hr (B.R.); sasa@hedera.hr (S.R.); 8 Faculty of Pharmacy and Biochemistry, University of Zagreb, Ante Kovacica 1, 10000 Zagreb, Croatia; ikosalec@pharma.hr; 9 Division of Molecular Medicine, Ruđer Bošković Institute, Bijenička cesta 54, 10000 Zagreb, Croatia; josipa.vlainic@irb.hr

**Keywords:** propolis, extracts, ethanol, PEG400, antioxidant activity, antimicrobial activity

## Abstract

We compared the chemical composition, antioxidant and antimicrobial activity of two propolis extracts: one obtained with nonaqueous polyethylene glycol, PEG 400 (PgEP), and the other obtained with ethanol (EEP)**.** We analyzed the total phenolic content (TPC) and the concentrations of ten markers of propolis antioxidant activity with HPLC-UV: caffeic acid, *p*-coumaric acid, *trans*-ferulic acid, *trans*-cinnamic acid, kaempferol, apigenin, pinocembrin, chrysin, CAPE, and galangin. Antioxidant activity was tested using DPPH and FRAP assay, and antimicrobial activity was assessed through minimum inhibitory concentrations (MICs) and minimum biofilm eradication concentration (MBEC) determination. Maceration gave the yield of propolis of 25.2 ± 0.08% in EEP, and 21.5 ± 0.24% in PgEP. All ten markers of antioxidant activity were found in both extracts, with all marker concentrations, except kaempferol, higher in EEP. There was no significant difference between the TPC and antioxidant activity of the PgEP and the EEP extract; TPC of PgEP was 16.78 ± 0.23 mg/mL, while EEP had TPC of 15.92 ± 0.78 mg/mL. Both extracts had antimicrobial activity against most investigated pathogens and *Staphylococcus aureus*, *Acinetobacter baumannii*, and *Escherichia coli* biofilms. EEP was more effective against all tested susceptible pathogens, except *E. coli*, possibly due to higher content of kaempferol in PgEP relative to other polyphenols. Nonaqueous PEG 400 could be used for propolis extraction. It gives extracts with comparable concentrations of antioxidants and has a good antioxidant and antimicrobial activity. It is a safe excipient, convenient for pediatric and veterinary formulations.

## 1. Introduction

Propolis is a honey bee product known for its antioxidant, antimicrobial, and anti-inflammatory properties [1,2]. Over the centuries, it had a significant role in traditional medicine and is still used today as an antibiotic alternative due to the growing problem of antimicrobial resistance [3]. Besides the antibiotic resistance problem, natural products are more valuable than synthetic ones due to their lower cytotoxicity [4].

Honey bees collect crude propolis from resinous plant material to strengthen wax combs and thermoinsulate the beehive, and at the same time, to protect the colony from pathogens. Various plants produce resinous compounds with anti-putrefaction, waterproofing, and heat-insulating properties to protect their plant organs. Honey bees gather these resins and transform them into propolis by chewing and mixing resins with salivary glands products and bee wax. Propolis (“bee glue”) is a strong adhesive for blocking holes and cracks in the beehive, strengthening the nest, and smoothing out the internal walls [2,3]. In addition, since incorporated into beehives and in direct contact with adult bees, it contains other compounds such as essential oils, pollen, enzymes from the honey bee’s digestive system, and other organic compounds [2].

**Figure 1:**
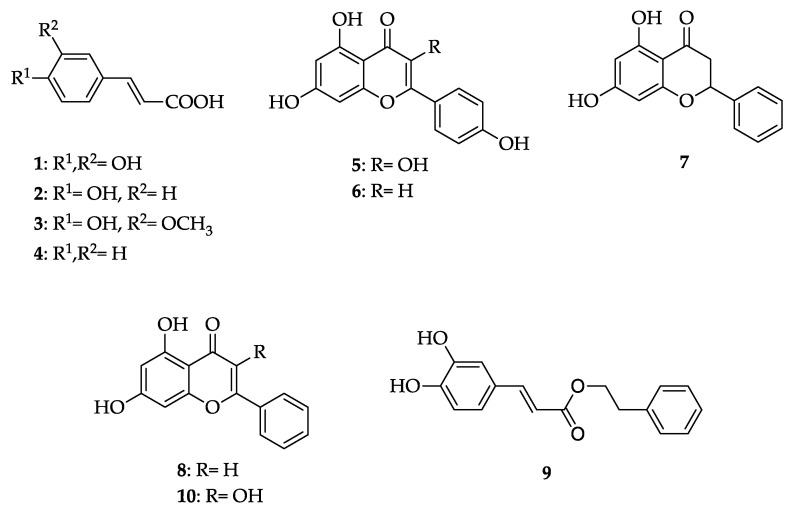
Ten bioactive molecules with antioxidant activity were used as representative markers of biological activity typical for poplar-type propolis: caffeic acid (**1**), *p*-coumaric acid (**2**), *trans*-ferulic acid (**3**), *trans*-cinnamic acid (**4**), kaempferol (**5**), apigenin (**6**), pinocembrin (**7**), chrysin (**8**), CAPE (**9**), galangin (**10**).

The composition of known bioactive molecules in propolis is not uniform but depends on the botanical origin, geographical area, season, bees’ genetics, beekeeping practices, and environmental factors [3,5,6,7]. Thus, the type of propolis is defined by its geographical and botanical origin and characterized by its representative markers previously identified in the literature [5,8,9,10]. For poplar-type propolis, typical are phenolic acids such as caffeic acid (**1**), *p*-coumaric acid (**2**), *trans*-ferulic acid (**3**), *trans*-cinnamic acid (**4**), kaempferol (**5**), apigenin (**6**), pinocembrin (**7**), chrysin (**8**), CAPE (**9**), and galangin (**10**) [9] (Figure 1). All these compounds are antioxidants and have antimicrobial activity. Naturally, the amount of these markers in propolis extracts will depend, among others, on the extraction method [3,11].

Extraction is a crucial and the most demanding stage in the utilization of bioactive compounds. Most in vitro assays of propolis biological activity use aqueous ethanolic (60–80% *w*/*w* EtOH) or 96% ethanolic (EtOH) extracts. Although very effective, these extracts are not suitable for all purposes. Ethanol is a relatively aggressive solvent, not appropriate for pregnant and breastfeeding women, children, certain patients, and veterinary medicine use. Furthermore, due to the relatively high content of beeswax in such extracts, they are not suitable for manufacturing various pharmaceutical, cosmetic, veterinary, and other products containing predominantly water. The latter acts as anti-solvent-type precipitation of beeswax when mixing with the rest of the water-based formulations. Thus, various methods and solutions are studied to replace conventional maceration with aqueous ethanol [12]. Although extraction technology is advanced and new solvents are introduced, ethanol remained a golden standard due to its efficacy and affordability [12].

Polyethylene glycol 400 (PEG 400) is a non-toxic, low-molecular-weight type of polyethylene glycol (PEG). It is widely used in different pharmaceutical formulations as an excipient serving as solvent, diluent, and humectant. Several studies compared the composition and activities of aqueous polyethylene glycol propolis extracts with ethanol, but they mostly used only 20% of PEG in water [13,14,15]. Pure, nonaqueous PEG 400 could be a more effective extraction solvent suitable for simple methods of propolis extraction such as maceration. Therefore, this study compared the chemical composition and biological activities of nonaqueous PEG 400 (PgEP) with the ethanolic propolis extract (EEP).

## 2. Materials and Methods

### 2.1. Propolis Collection and Pre-Treatment

Propolis was collected in Croatia from the beehives situated at ten stationary apiaries in Sisak-Moslavina County during one week in late summer. In the vicinity of apiaries, there are mixed oak and poplar tree forests. Samples of propolis were collected from two commercial traps per apiary. Each trap with a sample of the crude poplar-type propolis was chilled in a refrigerator at −20 °C for minimally 1 h, and harvested. Then, all collected propolis samples were mixed in one composite sample (1 kg), which was milled and prepared for extraction.

### 2.2. Propolis Extraction

#### 2.2.1. Propolis Extraction with 96% Ethanol as the Extraction Solvent

The propolis extract was obtained by maceration. First, ethanol (96%; 70.00 g) was added to milled propolis (30.00 g). This mixture was left at room temperature for 72 h with periodical stirring. Then, the mixture was filtered, yielding 60.00 g of isolated liquid propolis extract EEP (85.7% *w*/*w* against the mass of starting extraction solvent). This process was repeated three times, and it was estimated that EEP had 25.2% ± of propolis in a deep brown-colored solution with an intensive propolis odor. 

The yield (% from theoretical yield) is expressed as weight (% *w*/*w*) percent of isolated liquid propolis extract against the mass of starting extraction solvent (EtOH, PEG).

#### 2.2.2. Propolis Extraction with PEG 400 as the Extraction Solvent

Milled propolis (30.00 g) was mixed with PEG 400 (70.00 g). The obtained mixture was left at room temperature to macerate for 72 h with periodical stirring and filtration at the end. The process was repeated three times. This yielded 55.00 g (78.6% *w*/*w*) of liquid propolis extract PgEP with approximately 21.5% ± 0.24 propolis, in the form of a deep brown viscous liquid of intensive propolis odor.

### 2.3. Total Amount of Phenolic Compounds Determination

The total amount of phenolic compounds (TPC) was determined using the Folin–Ciocalteu (F–C) assay. First, a 200 μL of 10% F–C reagent was added to 100 μL of each propolis extract and vortexed thoroughly. Then, 800 μL of 700 mM Na_2_CO_3_ was added to each tube and incubated at room temperature for 2 h. 200 μL of each sample was transferred to a clear 96-well microplate, and the absorbance was read at 765 nm. A standard curve was calculated from the blank-corrected A_765_ of the gallic acid standards (c (gallic acid) = 0.05–0.5 mg/mL). Total phenolics were calculated as gallic acid equivalents using the regression equation between gallic acid standards and A_765_. 

### 2.4. High-Performance Liquid Chromatography Analysis with UV Detection (HPLC—UV–Vis) of Propolis Extracts

Quantitative analysis on ten representative markers (Figure 1) was performed by high-performance liquid chromatography (HPLC) with UV detection, using the modified method [9]. The samples (100 μL) were diluted prior to analysis with ethanol: water mixture, 75:25, *v*/*v* (900 μL), in ratio 1:10 *w*/*w* (10× dilution). Analyses were carried out on a Shimadzu LC201CHT instrument (Shimadzu Corporation, Kyoto, Japan), equipped with an autosampler, pump, degasser, column oven, and UV–Vis detector, under the following conditions: chromatographic column: Ascentis express (Supelco Inc., Bellefonte, PA, USA); C_18_; dimensions: 15 cm × 3.0 mm; diameter of particles in the column: 2.7 μm; mobile phase: A = 0.1% formic acid aqueous solution, B = methanol; gradient: 0 min, 80% A, 20% B; 3 min, 70% A, 30% B; 60 min, 20% A, 80% B; 90 min, 20% A, 80% B; 100 min, 70% A, 30% B; 105 min, 80% A, 20% B; column temperature: 30 °C; flow: 0.25 mL/min; analysis time: 110 min; wavelength on UV–Vis detector: for detection: 370 nm, for integration: 290 nm; injection volume: 10 μL; pressure: 210–290 bars.

### 2.5. Antioxidant Assays of Propolis Extracts

#### 2.5.1. Determination of Ferric Reducing/Antioxidant Power (FRAP Assay)

A solution of 10 mM 2,4,6-Tri (2-pyridyl)-s-triazine (TPTZ) and 20 mM ferric chloride was diluted in 300 mM sodium acetate buffer (pH 3.6) at a ratio of 1:1:10. Extracts (20 μL) were added to the 96-well microplate, followed by a working FRAP solution (280 μL). The mixture was shaken and incubated for 30 min at 37 °C in the dark. The final concentration of tested extracts was 1 mg/mL. The absorbance at 593 nm was recorded using microplate reader μQuant^TM^ (BioTek^®^ Instruments, Inc., Winooski, VT, USA). Ferrous sulfate (FeSO_4_ · 7H_2_O) was used to develop a 20–2000 μmol/L standard curve. All results were then expressed as Fe^2+^ equivalents (Fe^2+^ μmol/mg propolis extract). All tests were carried out in triplicate, and the results were averaged.

#### 2.5.2. DPPH Radical Scavenging Assay

An equal volume of tested phenolic extracts at various concentrations was added to a solution of 2,2-diphenyl-1-picrylhydrazyl (DPPH; final concentration 100 μM in absolute ethanol), in a 96-well microtiter plate. An α-tocopherol solution of 10 mg/mL was a positive control, and ethanol was used as a negative control. After the incubation of 30 min in the dark, the absorbance was recorded at 517 nm on μQuant^TM^ (BioTek^®^ Instruments, Inc., Winooski, VT, USA) reader at room temperature. All measures were carried out in triplicate.

The percentage scavenging of test samples at each concentration was calculated using the following formula:

[(Abs_control_ − Abs_compound_)/Abs_control_] × 100

The EC_50_ values for each compound were calculated from dose-response curves using linear regression analysis.

### 2.6. Determination of the Antimicrobial Activity of Propolis Extracts

#### 2.6.1. Minimal Inhibitory Concentration (MIC) on Model Pathogenic Microorganisms

Antimicrobial efficacy of propolis extracts PgEP and EEP was tested under in vitro conditions on the American Type Culture Collection (ATCC) strains of the following model pathogenic microorganisms (M): *Staphylococcus aureus* (ATCC 29293), methicillin-resistant *Staphylococcus aureus* (MRSA) (MFBF collection), methicillin-sensitive *Staphylococcus aureus* (MSSA) (MFBF collection), *Enterococcus faecalis* (ATCC 9212), *Enterococcus faecalis* (vancomycin-resistant enterococci, VRE, MFBF collection) (M5); *Escherichia coli* ATCC 10536 (M6); *Acinetobacter baumannii* (ATCC 43498), *Pseudomonas aeruginosa* ATCC 9027, and *Candida albicans* (ATCC 9002). All tested species denoted with MFBF were from the collection of the Department of Microbiology, Faculty of Pharmacy and Biochemistry, University of Zagreb, Zagreb, Croatia.

The serial microdilution procedure was used to determine the MIC of the extracts. Cell suspensions were prepared from the parent culture in phosphate-buffered saline (PBS) buffer (pH 7.4), and these were adjusted to 0.5 McFarland units by nephelometry. The testing was performed in serial dilution in 96-well microtiter plates in the range from 100 to 0.7125 μg/mL, by addition of 100 μL of the solution of propolis extract dissolved in Mueller Hinton broth. After inoculation 100 μL of each bacterial culture adjusted to 10^5^ cfu/mL, plates were incubated for 24 h at 37 °C. The MIC values were determined by the addition of 10 μL of 0.5 mg/mL solution of 2,3,5-triphenyl-2*H*-tetrazolium chloride (TTC; redox indicator) per single well, and, after incubation for 4 h at 30 °C, the absorbance was determined by spectrophotometry at wavelength 490 nm. The MIC values were expressed as the propolis extract concentration at which 80% reduction in bacteria occurred (MIC_80_). For fungal species, the MIC values were determined in the RPMI medium with additional glucose, using the same scheme as with bacteria. After incubation (48 h, 37 °C, aerobic conditions, in the dark), 2,3-bis(2-methoxy-4-nitro-5-sulfophenyl)-5-carboxanilide-2*H*-tetrazolium) (XTT; redox indicator) was added in combination with menadion, and the absorbance was determined by spectrophotometry at wavelength 540 nm. The MIC values were determined as the propolis extract concentration at which 80% reduction in bacteria or fungi occurred (MIC_80_). The negative control contained only the medium and the solvent (without added microorganisms and propolis), while the positive control was exposed to the influence of antibiotics (gentamicine sulphate, norfloxacin, colistin, nystatin) and voriconazole (Pfizer, New York, NY, USA) as antifungal agent (MIC for *C. albicans*, ATCC 90028, was 0.01 ug/mL).

#### 2.6.2. Determination of Minimum Biofilm Eradication Concentrations (MBEC) of Propolis Extracts

We determined the minimum biofilm eradication concentration (MBEC) of PgEP and EEP on *S. aureus*, *E. coli*, *A. baumannii*, and *C. albicans*. Wells (96 well plate) were pretreated with FBS (fetal bovine serum; Millipore Sigma, St. Louis, MI, USA) (250 µL per well). In each well, 100 µL of bacterial (10^7^ CFUs/mL) or yeast (5 × 10^6^ CFUs/mL) suspension was added. Negative controls contained broth only, and gentamycin and amphotericin were used for positive controls. Plates with bacteria were covered and incubated for 24 h, while plates with yeasts were left for 48 h in aerobic environment. After incubation, we aspirated each well. In order to remove all non-adherent bacteria/yeast, each well was washed three times and vigorously shaken. The attached cells that remained were fixed with methanol (15 min), and left overnight to dry. We used 1% crystal violet to stain the formed biofilm for 5 min. The remaining stain was rinsed by placing the plate under running tap water and the plates were left to dry. Adherent cells were solubilized using ethanol. The absorbance was read at 570 nm. The MBEC value represents the lowest dilution of a compound at which bacteria fail to grow.

### 2.7. Statistical Analyses

All extractions and analyses were carried out in triplicates. Mirobiological analyses were performed as technical and biological replicates. Analyses were carried out using GraphPad Prism software 9.1.1. for Windows (GraphPad Software, San Diego, CA, USA, www.graphpad.com, accessed on 2 January 2021). MIC/MBEC_80_ were obtained using non-linear regression. The normality of distributions were confirmed with the D’Agostino-Pearson omnibus test and Shapiro–Wilk test. Unpaired *t*-test was used to test the differences between the two corresponding groups, with the significance set at *p* ≤ 0.05.

## 3. Results

**Figure 2:**
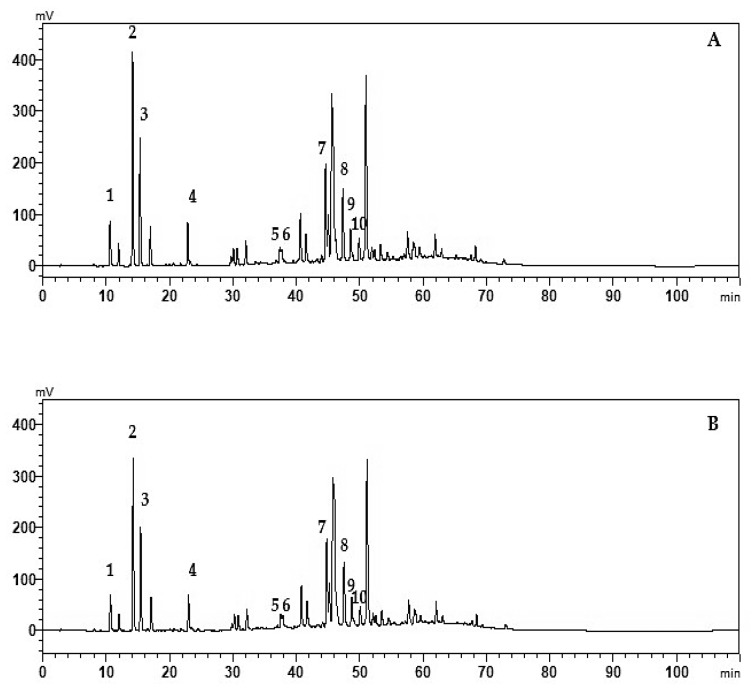
Chromatograms of propolis extracts; (**A**) EEP, (**B**) Pg-EP, with representative markers of activity: (1) caffeic acid, (2) *p*-coumaric acid, (3) *trans*-ferulic acid, (4) *trans*-cinnamic acid, (5) kaempferol, (6) apigenin, (7) pinocembrin, (8) chrysin, (9) CAPE, (10) galangin.

**Figure 3:**
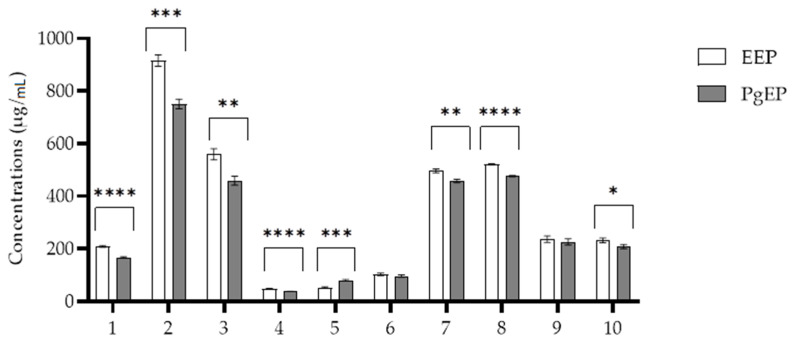
Concentrations of active substances (means ± SD) found in both poplar-type propolis extracts: (1) caffeic acid, (2) *p*-coumaric acid, (3) *trans*-ferulic acid, (4) *trans*-cinnamic acid, (5) kaempferol, (6) apigenin, (7) pinocembrin, (8) chrysin, (9) CAPE, (10) galangin. Statistically significant differences are marked as * for *p* ≤ 0.05, ** for *p* ≤ 0.01, *** for *p* ≤ 0.001, and **** for *p* ≤ 0.0001. Unpaired *t*-test was used to test the differences between the two corresponding groups.

Representative markers of antioxidant activity (Figure 1) were found in both extracts, as presented with chromatograms in Figure 2 and their concentrations in Figure 3. In comparison to Pg-EP, EEP had higher concentrations of all markers except kaempferol (5).

**Figure 4:**
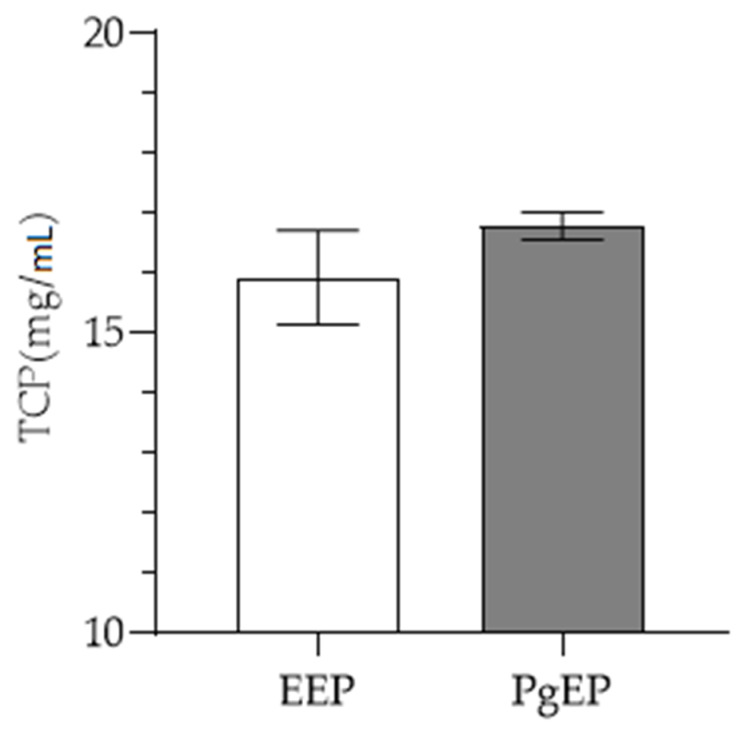
Total amount of phenolic compounds (means ± SD) in EEP and PgEP.

**Figure 5:**
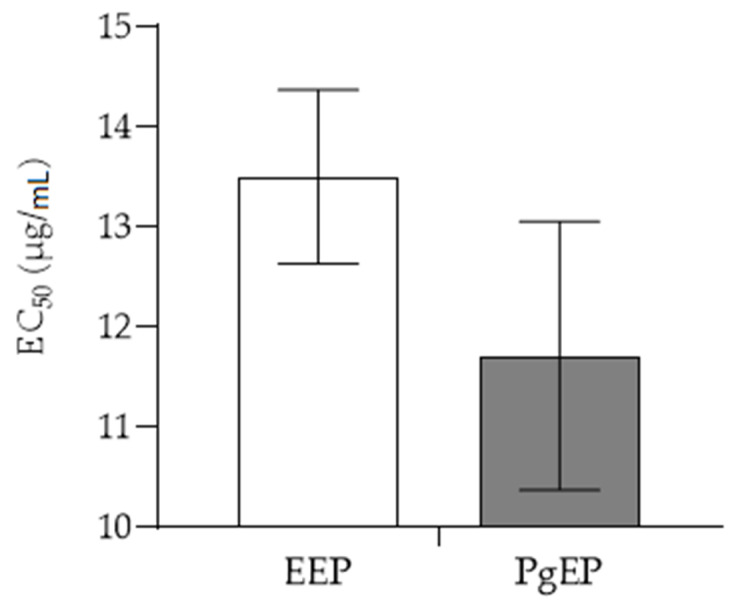
DPPH assay (mean values ± SD) of ethanolic (EEP) and PEG 400 propolis extract (Pg-EP).

**Figure 6:**
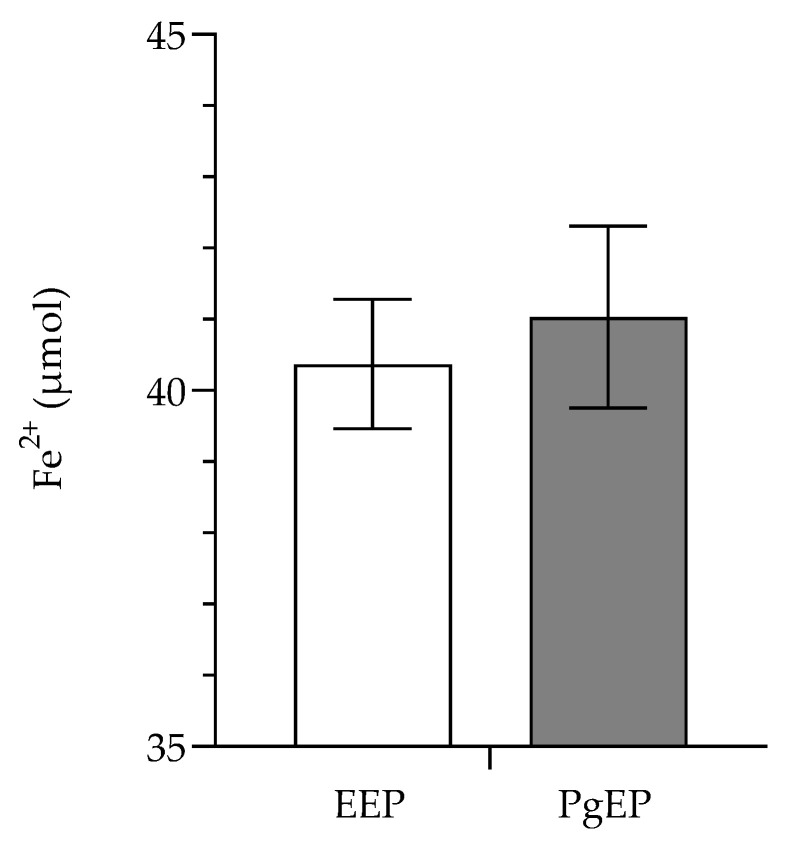
FRAP assay (mean values ± SD) of ethanolic (EEP) and PEG 400 propolis extract (Pg-EP).

Total amount of phenolic compounds (TPC) is presented in Figure 4, while antioxidant activities of EEP and PgEP are presented in Figure 5 and Figure 6. There was no significant difference between the TPC and antioxidant activity of the PgEP and the EEP extract. TPC of PgEP was 16.78 ± 0.23 mg/mL, while EEP had TPC of 15.92 ± 0.78 mg/mL (Figure 3). In DPPH assay, EC_50_ was 11.71 ± 1.34 µg/mL for PgEp vs. 13.50 ± 0.87 µg/mL for EEP. In the FRAP assay, the reducing ability of PgEP was 41.03 ± 0.91 vs. 40.37 ± 1.28 Fe^2+^ µmol/mg propolis extract of EEP (Figure 5 and Figure 6).

Antibacterial activities of EEP and PgEP are presented in Table 1 and Table 2. MIC_80_ values are expressed as the amount (%) of initial propolis content in the extract. Both extracts had antimicrobial activity against *S. aureus*, MRSA, MSSA, *E. faecalis*, *E. coli* and *A. baumannii*, but not against VRE, *P. aeruginosa*, and *C. albicans* (Table 1). In addition, the extracts had good activity against biofilm formed by *S. aureus*, *E. coli*, and *A. baumannii*, but did not work against *C. albicans* biofilm (Table 2). The most sensitive bacteria to EEP were gram positives, especially *S. aureus* and MSSA. EEP was more effective, with 40× dilution reducing 80% of *S. aureus*, compared with PgEP, with effective 10× dilution. The remarkable difference was in reducing MSSA, where even 52.5× diluted EEP was effective as 10× diluted PgEP. EEP was not more effective than PgEP against *E. coli*, which was interestingly the most susceptible bacteria to antimicrobial activity of PgEP.

## 4. Discussion

Of all the honey bee products, propolis is the most potent antioxidant [16]. We chose 10 representative markers of propolis antioxidant activity typical for poplar propolis (Figure 1) [9]. These are all polyphenols: caffeic acid (1), *p*-coumaric acid (2), *trans*-ferulic acid (3), *trans*-cinnamic (4), and CAPE (9) are phenolic acids and derivatives, while kaempferol (5), apigenin (6), pinocembrin (7), chrysin (8), and galangin (10) are flavonoids (Figure 1). Propolis extracts inhibit the generation of reactive oxygen species (ROS) through nuclear factor E2-related factor 2 (Nrf2) and activation of antioxidant response element (ARE), responsible for transcription of antioxidant enzymes [17,18]. This mechanism of antioxidant action has also been confirmed for all the chosen representative markers [19,20,21,22,23,24,25,26,27,28]. Undoubtedly, it results from certain polyphenols synergy, and this synergy will depend on the method of propolis extraction [16].

As the technology develops, so do the methods of propolis extraction. In recent years, progress has been made toward more efficient methods regarding the time and solvents used. These methods include Soxhlet, ultrasound-assisted extraction (UAE), microwave-assisted extraction (MAE), supercritical CO_2_ extraction (scCO_2_), and high-pressure methods [8]. Still, maceration is the most affordable and accessible extraction method, with ethanol as the relatively most effective solvent. Although there are some new promising solvents, such as natural deep eutectic solvents (NADES) [8], most of the further developed technologies are still using ethanol as the solvent. Ethanolic and aqueous ethanolic propolis extracts are used commercially in most propolis-based products and various in vitro research applications. The highest yields of propolis extract is obtained by aqueous ethanol containing 70 to 96% of ethanol [8]. In addition, Ma et al. (2016) confirmed that ethanol and methanol propolis extracts had greater antioxidant activity than water, ethyl acetate, chloroform, and benzene extracts [29]. 

On the other hand, several studies showed the contrary. Water extracts of Brazilian and Turkish propolis demonstrated greater scavenging capabilities of DPPH, H_2_O_2_, O_2_^•−^, and ^•^OH radicals [30]. Rocha et al. (2013) demonstrated that differently prepared extracts of propolis had significant DPPH radical scavenging activity. Still, water extract exhibited a higher antioxidant activity than EEP [31]. Galeotti et al. (2018) demonstrated that propolis from the same source, in the form of aqueous ethanolic, glycolic (1,2-PG), glyceric, oil solutions, and powder extract had a similar chemical composition with differences in TPC [32]: the highest was in 1,2-PG extract (81.2 ± 3.7%), followed by hydroalcoholic extract (69.7 ± 2.0%), while the lowest was in powder, a micronized sample composed of propolis with a minimum of 12% total polyphenols. This form had the highest concentrations of caffeic (1), *p*-coumaric (2), ferulic (3), and isoferulic acids. All extracts had comparable antioxidant activity, presented as μg Trolox equivalent/mg polyphenols [32].

To the best of our knowledge, the use of PEG as the extraction solvent was mentioned for the first time among other organic solvents by Sosnowski (1981), in his patent. [33]. However, there are no data on the antioxidant’s content and activity presented in the patent. Later, other authors used aqueous PEG 400 (AqPg-EP, with 20% PEG 400), but these extracts had lower concentrations of bioactive molecules and antioxidant and antimicrobial activity [13,14,34]. Interestingly, none of the previous studies used the pure, nonaqueous PEG 400 for propolis extraction. According to Kubiliene et al. (2015), it is more difficult to sterilize the extract by filtration when PEG 400 content in the solvent is above 20% [13]. Still, the methods of propolis extract sterilization should be a subject of further research.

In a recent study by Liaudanskas et al. (2021), TPC in AqPgEP (400.36 µg/mL) was similar to that of EEP (433.53 µg/mL), but AqPgEP had a much lower concentration of apigenin (6.5 vs. 13.7 µg/mL), galangin (0.08 vs. 1.12 µg/mL), and kaempferol (2.0 vs. 6.0 µg/mL) [34]. AqPgEP antioxidant activity was 12 times lower than the activity of EEP when measured by DPPH (107.1  ±  10.1 vs. 1299.5  ±  43.9 TE/g), while the FRAP assay showed half the activity of AqPgEP in comparison to EEP (10.7  ±  1.2 vs. 21.3  ±  4.2 TE/g) [34]. Kubiliene et al. (2015, 2018) also used aqueous polyethylene glycol 400 [13,14]. They extracted propolis with 70% ethanol (EEP), water (AqEP), 20% PEG 400 and water (AqPgEP), olive oil (oEP), 20% PEG 400, and a mixture of olive oil and water (50/50) (oAqPgEP). The TPC of EEP was similar to that of the extract with AqPgEP (12.7 mg/mL vs. 10.7 mg/mL GAE). Kaempferol and galangin, found in EEP, were not identified in the first three extracts, while they were found in minimal amounts in oAqPgEP [13]. The AqPgEP had the highest radical scavenging activity, while the oEP had the lowest activity [13]. In their later study [14], the amount of TPC was 10 and 17 times higher in EEP than AqPgEP and AqEP, respectively. AqPgEP had twice more TPC than AqEP. EEP was shown to have the greatest ROS scavenging capacity, while AqPgEP had twice the capacity of AqEP [14].

**Figure 7:**
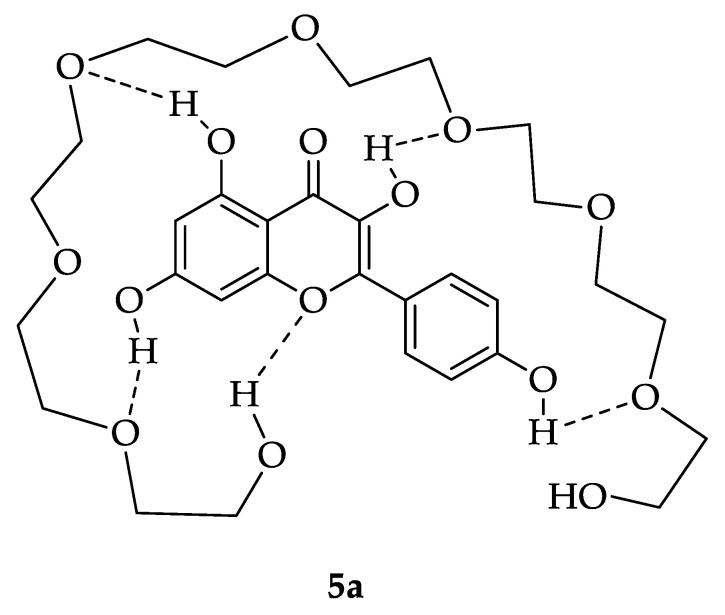
Proposed possible structure of molecular complex 5a of kaempferol (5) and polyethyleneglycol 400 (PEG 400) in PEG 400 propolis extract (PgEP) which is stabilized by five hydrogen bonds. The latter additionally explains significantly increased chemoselectivity of its extraction in PgEP over kaempferol content in the ethanolic propolis extract (EEP) obtained with EtOH as the extraction solvent.

In our study, all the ten markers of activity were found in both extracts. There was no significant difference between the TPC (Figure 3) and the antioxidant activity of the PgEP and the EEP extract (Figure 5 and Figure 6). In comparison to EEP, PgEP had lower concentrations of all marker compounds except kaempferol (5). This selectivity for kaempferol is a result of two facts:

(i) PEG 400 is a slightly more polar solvent than EtOH and more effectively extracts more polar flavonoid compounds. Since kaempferol (5) is the most polar propolis flavonoid of all analyzed, significantly higher quantitative flavonoid 5 content was obtained with PEG 400 than with less polar EtOH; 

(ii) Due to the presence of four phenolic hydroxyl (OH) groups which could act as hydrogen bond donors and one chromene oxygen acting as hydrogen bond acceptor, kaempferol (5) could form a kind of molecular complex with PEG 400, which would be stabilized by five (!) hydrogen bonds. PEG 400, with its average molecular weight (Mw) 380–420, and average 8.7 ethylene oxide (EO) units per molecule, contains two hydroxyl (OH) groups, at the beginning and the end of the chain, as well as 7 or 8 oxygen atoms of ether functions. The structure of possible kaempferol-PEG 400 complex (5a), which might explain the significantly increased chemoselectivity of its extraction with PEG 400 in PgEP over the EEP, is given in Figure 7.

Since there is a correlation between phenolic levels of propolis extracts and their antioxidant and antimicrobial activities [7,13,35], we expected to see that both of our extracts had comparable antimicrobial activity. Tosi et al. [36] were among the first to prove that solvents used for propolis extraction can increase the antimicrobial efficacy of propolis. Their propolis oil extracts had a wider range of antimicrobial activity than ethanol (60%), propylene glycol, and glycerine, but ethanol and propylene glycol extracts had good activity against yeasts [36]. In the previously mentioned study by Kubiliene et al. (2015), propolis extracts made only with water or oil had no antimicrobial effect [13]. EEP and non-alcoholic extracts with PEG 400 (AqPgEP and oAqPgEP) had antimicrobial activity against *S. aureus*, *E. coli*, *P. aeruginosa*, *K. pneumoniae*, *B. cereus*, and *C. albicans.* Interestingly, the antimicrobial activity of propolis non-alcoholic extracts (AqPgEP and oAqPgEP) against all investigated microorganisms was equal or even higher (against *P. aeruginosa* and *K. pneumoniae*) than EEP [13]. In our study, EEP was more effective against most of the investigated pathogens except *E. coli.* This could be due to the chemoselective extraction of kaempferol because it was shown that kaempferol is one of the strongest flavonoid inhibitors of *E. coli* growth [37]. In that same study carried out by Wu et al. (2013), kaempferol was located deeply in the hydrophobic core of the lipid bilayer of the model membrane, decreasing the membrane fluidity most while exhibiting the highest antibacterial activity against *E. coli* in comparison to other 10 commercially available flavonoids [37].

Our findings are consistent with studies that confirmed the superior antimicrobial efficacy of ethanolic extracts in comparison to solvents such as hexane [38], water [13,39,40,41], and propylene glycol [40]. In their comprehensive literature review on antimicrobial properties of propolis, Przybyłek and Karpiński (2019) sort out the antibacterial activity of propolis extracts prepared with different solvents [42]. In almost every pathogen studied (even the *E. coli*) EEP had lower MIC levels than water extracts. On the other hand, organic solvents such as dichloromethane and methanol propolis extracts were more effective than EEP [42]. However, these solvents are quite toxic and impose a health risk to anyone working with them.

## 5. Conclusions

Even though PEG 400 gave a lower yield of propolis extraction than ethanol (21.5 ± 0.24 vs. 25.2 ± 0.08%), it gave very similar TPC and antioxidant activity. Moreover, it extracted all the ten markers of antioxidant activity with chemoselectivity over kaempferol. This could also explain the greater activity of PEG 400 propolis extract against *E.coli* in comparison to ethanolic extract. PEG400 is a polar, hydrophilic solvent known as a non-toxic, which is very well-tolerated and of low cost. It is acceptable even in the most demanding cases, such as pediatric formulations. With the advent of new green methods of polyethylene glycol production [43], we believe this will become a sustainable and environmentally friendly solvent base for propolis extraction.

## 6. Patents

Some of the presented methods and results are published in the WIPO patent No. WO/2020/169425: LIQUID PROPOLIS EXTRACT, ITS FORMULATION AND USE THEREOF [44]: extraction method, HPLC analyses, MIC determination.

## Figures and Tables

**Table 1:** MIC_80_ values of ethanolic (EEP) and PEG 400 propolis extract (Pg-EP), expressed as % of propolis.

No.	MIC (%)	EEP	PgEP
1	*S. aureus* ATCC 29293	0.63	2.15
2	MRSA MFBF collection	1.26	2.15
3	MSSA MFBF collection	0.48	2.15
4	*E. faecalis* ATCC 9212	2.52	12.42
5	*E. faecalis* VRE MFBF collection	>25	21.5
6	*E. coli* ATCC 10536	2.52	1.98
7	*A. baumannii* ATCC 43498	1.26	2.15
8	*P. aeruginosa* ATCC 9027	>25.2	>21.5
9	*C. albicans* ATCC 90028	>25.2	>21.5

**Table 2:** Biofilm (minimal biofilm eradication concentration) of ethanolic (EEP) and PEG 400 propolis extract (Pg-EP), expressed as % of propolis.

No.	MBEC (%)	EEP	PgEP
1	*S. aureus* ATCC	0.630	2.15
2	*E. coli*	2.52	1.98
3	*A. baumannii*	1.26	2.15
4	*C. albicans*	>25.2	>21.4

## Data Availability

Data are presented in the manuscript.
